# Endoscopic transorbital extradural anterior clinoidectomy: A stepwise surgical technique and case series study [SevEN-013]

**DOI:** 10.3389/fonc.2022.991065

**Published:** 2022-08-29

**Authors:** Jaejoon Lim, Kyoung Su Sung, Jihwan Yoo, Jiwoong Oh, Ju Hyung Moon

**Affiliations:** ^1^ Department of Neurosurgery, Bundang CHA Medical Center, CHA University College of Medicine, Seongnam, South Korea; ^2^ Department of Neurosurgery, Dong-A University Hospital, Dong-A University College of Medicine, Busan, South Korea; ^3^ Department of Neurosurgery, Brain Tumor Center, Gangnam Severance Hospital, Yonsei University College of Medicine, Seoul, South Korea; ^4^ Department of Neurosurgery, Severance Hospital, Yonsei University College of Medicine, Seoul, South Korea; ^5^ Department of Neurosurgery, Endoscopic Skull Base Center, Severance Hospital, Yonsei University College of Medicine, Seoul, South Korea

**Keywords:** anterior clinoid process, anterior clinoidectomy, cadaveric study, endoscopic transorbital approach, extradural technique

## Abstract

**Background:**

Anterior clinoidectomy is an important procedure for approaching the central skull base lesions. However, anterior clinoidectomy through the endoscopic transorbital approach (ETOA) still has limitations due to technical difficulties and the structural complexity of the anterior clinoid process (ACP). Therefore, the authors designed a stepwise surgical technique of extradural anterior clinoidectomy through the ETOA. The purpose of this study was to evaluate the feasibility of this technique.

**Methods:**

Anatomical dissections were performed in 6 cadaveric specimens using a neuroendoscope and neuro-navigation system. The extradural anterior clinoidectomy through the ETOA was performed stepwise, and based on the results, this surgical technique was performed in the 7 clinical cases to evaluate its safety and efficiency.

**Results:**

Endoscopic extradural anterior clinoidectomy was successfully performed in all cadaveric specimens and patients using the proposed technique. This 5-step technique enabled detachment of the lesser wing of sphenoid bone from the ACP, safe unroofing of the optic canal, and resection of the optic strut without injuring the optic nerve and internal carotid artery. Since the sequential resection of the 3 supporting roots of the ACP was accomplished safely, anterior clinoidectomy was then successfully performed in all clinical cases. Furthermore, no complications related to the anterior clinoidectomy occurred in any clinical case.

**Conclusion:**

We designed a stepwise surgical technique that allows safe and efficient anterior clinoidectomy through the ETOA. Using this technique, extradural anterior clinoidectomy can be accomplished under direct endoscopic visualization with low morbidity. Since this technique is applicable to the central skull base surgery where anterior clinoidectomy is necessary, it expands the application of the ETOA.

## 1 Introduction

Endoscopic brain tumor surgery is becoming increasingly accessible, and with the development of the endoscopic transorbital approach (ETOA), it has been welcomed along with the endoscopic endonasal approach (EEA) (1–5). To date, most applications of the ETOA have been for lesions in the temporal and middle fossa region (6, 7). Although using the ETOA for extra-axial lesions such as trigeminal schwannoma or sphenoid wing meningioma would be advantageous, efforts have also been made for intra-axial lesion tumors in the temporal lobe and insular along the ETOA trajectory (6, 8–10). Additionally, recent attempts have been made to extend the use of the ETOA in the anterior or the posterior fossa (11–13). The anterior clinoid process (ACP) is an important landmark in anterior skull base surgeries (14–16). In classic transcranial surgeries, anterior clinoidectomy is a vital procedure to remove tumors in the skull base region, and various methods are undergoing research (17–24). However, anterior clinoidectomy through the ETOA remains challenging due to technical difficulties and structural complexity. Since the lesser wings of the sphenoid bone (one of the 3 supporting roots of the ACP) are removed by the craniotomy in the ETOA, it offers certain advantages in anterior clinoidectomy. Nevertheless, the issue lies with the difficulty in safe resection of the other supporting roots, including the roof of the optic canal and optic strut. Because the anatomy around the ACP under the surgical view of ETOA is generally unfamiliar for neurosurgeons, the preservation of critical adjacent neurovascular structures including the optic nerve and internal carotid artery (ICA) during endoscopic transorbital extradural anterior clinoidectomy is complicated.

Here, we present the anatomical considerations and surgical technique of endoscopic transorbital extradural anterior clinoidectomy in a stepwise manner. We verified the feasibility of this technique by a cadaveric study, and its clinical significance through a case series.

## 2 Materials and methods

### 2.1 Cadaveric study

This study was approved by the cadaveric study committee of the Yonsei University of Medicine. Here, the twelve sides of the six cadaveric heads were used. Thiel embalming and ethanol-glycerin fixation were used to prepare the cadaveric heads, which were then injected with silicone rubber injection compounds (MICROFIL^®^; Flow Tech, Inc., Carver, MA, USA) to fill and opacify the blood vessels. The cadaveric heads were scanned with computed tomography (CT) prior to dissection, and the pictures were transferred to a cranial navigation system platform (Stryker navigation system, 2825 Airview Boulevard, Kalamazoo, MI 49002, USA). The neuro-navigation system (Stryker navigation system, 2825 Airview Boulevard, Kalamazoo, MI 49002, USA) determined the target spot and surgical corridor. All anatomical dissections occurred at the Yonsei University College of Medicine’s surgical anatomy laboratory and Severance Hospital. The dissections were carried out using a rigid endoscope with a diameter of 4 mm and a length of 18 cm, as well as 0° and 30° optic lenses (Stryker neuroendoscopy, 2825 Airview Boulevard, Kalamazoo, MI 49002, USA). Endoscopic transorbital extradural anterior clinoidectomy with a 5-step technique was performed on both sides of the heads. ETOAs were performed with or without the orbital rim resection to compare the accessibility of each approach to ACP. The anatomy around the ACP was explored under the surgical view of the ETOA. The 3D reconstruction model for the anatomical structures was made using Osirix software (25).

### 2.2 Clinical cases

After analyzing the results of the cadaveric procedure, stepwise endoscopic transorbital extradural anterior clinoidectomy was performed in seven clinical cases by the same neurosurgeon. The patients were retrospectively analyzed from January 2021 to December 2021.

## 3 Results

### 3.1 Three roots of the ACP

**Figure 1:**
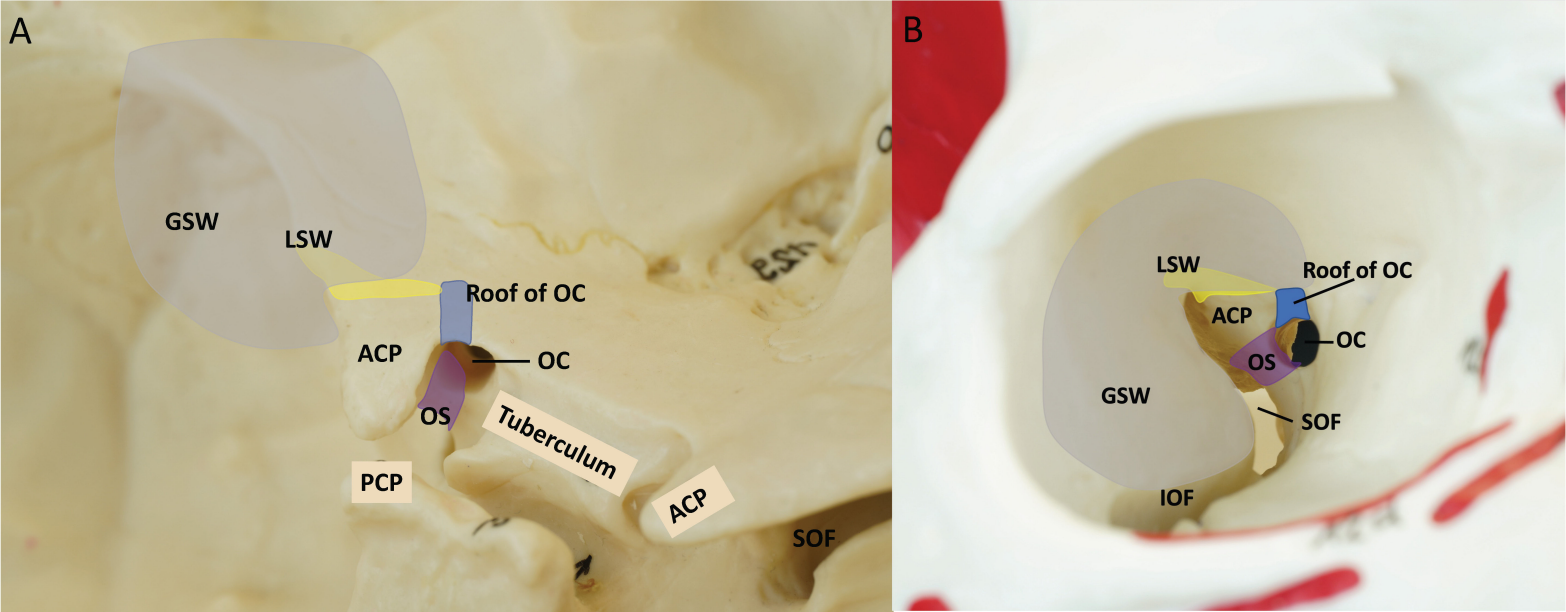
Three supporting roots of the ACP. Yellow = lesser wing of sphenoid bone (Lateral root), Blue = the roof of the optic canal (anterior root), Purple = optic strut (posterior root), Gray = craniotomy of endoscopic transorbital approach. **(A)** Left oblique view of the anterior bony skull base **(B)** Right intra orbital view. ACP, anterior clinoidal process; GSW, greater wing of sphenoid bone; IOF, inferior orbital fissure; LSW, lesser wing of sphenoid bone; OC, optic canal; OS, optic strut; PCP, posterior clinoidal process; SOF, superior orbital fissure.

The ACP has the structural complexity of bony anatomy (26–29). The anterior clinoid process is anatomically connected to the surrounding skull through 3 roots (**Figure 1**) (30). The lateral root of the ACP is most medial part of the lesser sphenoid wing (LSW), and the anterior root is the roof of the optic canal. The posterior root of the ACP is the optic strut. The ACP borders the sphenoid bone, superior orbital fissure, optic nerve, and ICA. To achieve successful anterior clinoidectomy, the three roots of the ACP (LSW, roof of the optic canal, and optic strut) should be safely removed without damaging the surrounding structures.

### 3.2 Endoscopic transorbital extradural anterior clinoidectomy in a stepwise manner

**Figure 2:**
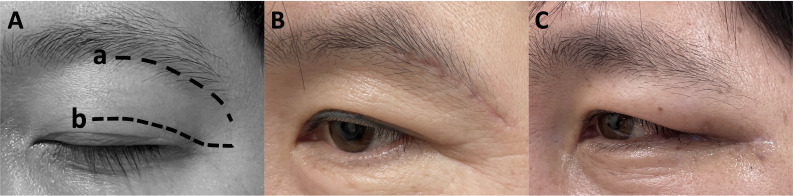
**(A)** Comparison of skin incision using a; below-the-eyebrow technique and b; transeyelid technique. **(B, C)** Postoperative patient photographs showing aesthetic results 1 month after ETOA surgery using each technique. B; below-the-eyebrow technique, C; below-the-eyebrow technique. ETOA, endoscopic transorbital approach.

The ETOA surgery was performed as described previously in the literature (7, 8, 11, 31). Skin incision was made using either trans eyelid or below-the-eyebrow techniques (**Figure 2**). The periosteum was cut after identification of the orbital rim, and dissection in the sub periorbital plane was performed till the superior orbital fissure (SOF) and inferior orbital fissure (IOF) was reached. Also, lateral orbital rim osteotomy or superior-lateral orbital rim (SLOR) osteotomy was performed selectively.

#### 3.2.1 Step 1

**Figure 3:**
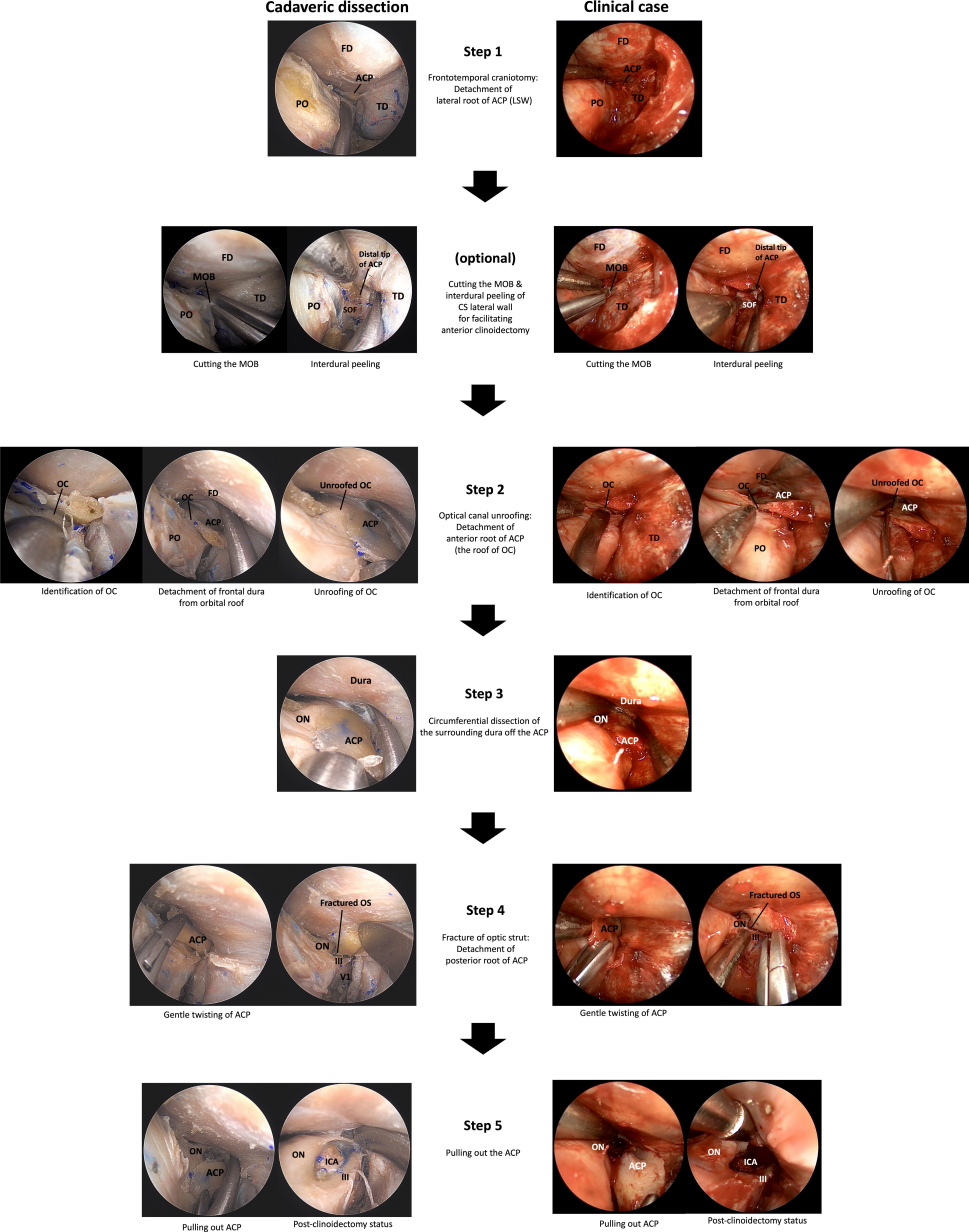
A 5-step technique for the endoscopic transorbital extradural anterior clinoidectomy. ACP, anterior clinoidal process; CS, cavernous sinus; FD, frontal dura; ICA, internal carotid artery; III, oculomotor nerve; IOF, inferior orbital fissure; LSW, lesser wing of sphenoid bone; MOB, meningo-orbital band; OC, optic canal; ON, optic nerve; OS, optic strut; PO, periorbita; SOF, superior orbital fissure; TD, temporal dura; V1, ophthalmic branch of trigeminal nerve.

First, the general procedure of ETOA, a frontotemporal craniotomy through ETOA was performed (**Figure 3**, **Video 1**). The greater sphenoid wing (GSW), LSW, and lateral part of frontal base bone were removed to expose the frontotemporal dura. Disconnection of the lateral root of the ACP can be achieved during resection of LSW.

#### 3.2.2 (optional)

**Figure 4:**
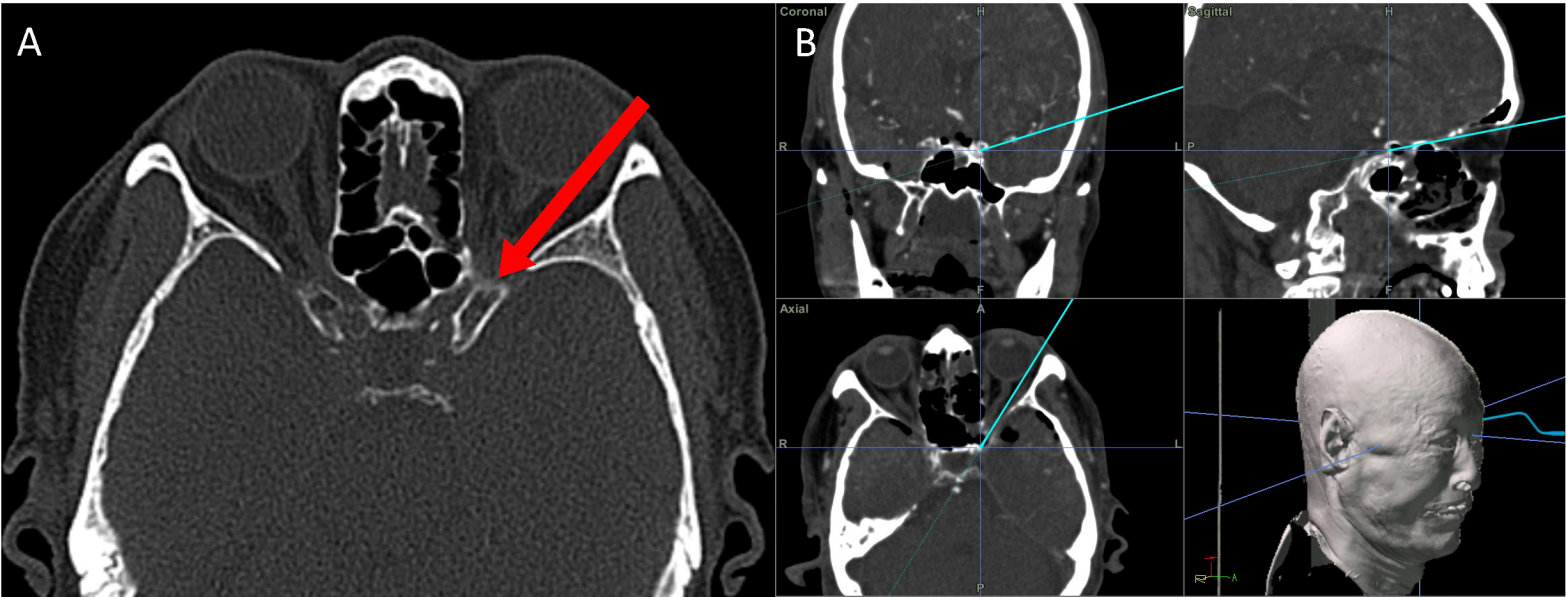
The direction for approaching the ACP through the corridor of ETOA corridor. **(A)** The surgical direction of ETOA (red arrow) is parallel to longitudinal direction of the ACP. **(B)** Coronal, sagittal and axial direction of ETOA in neuronavigation system. ACP, anterior clinoidal process; ETOA, endoscopic transorbital approach.

Cutting the meningo-orbital band (MOB) and peeling the outer dura from the lateral wall of the cavernous sinus (CS) can provide a better surgical view of the ACP, which facilitates anterior clinoidectomy through the ETOA. While this procedure is essential for anterior clinoidectomy by enabling access to the distal tip of the ACP; in the classic transcranial approach where the ACP is approached from the lateral to medial direction, anterior clinoidectomy is not mandatory in the ETOA because the surgical corridor in the latter is parallel to the longitudinal axis of the ACP and enables the surgeon to access its tip (**Figure 4**).

#### 3.2.3 Step 2 (optic canal unroofing)

**Figure 5:**
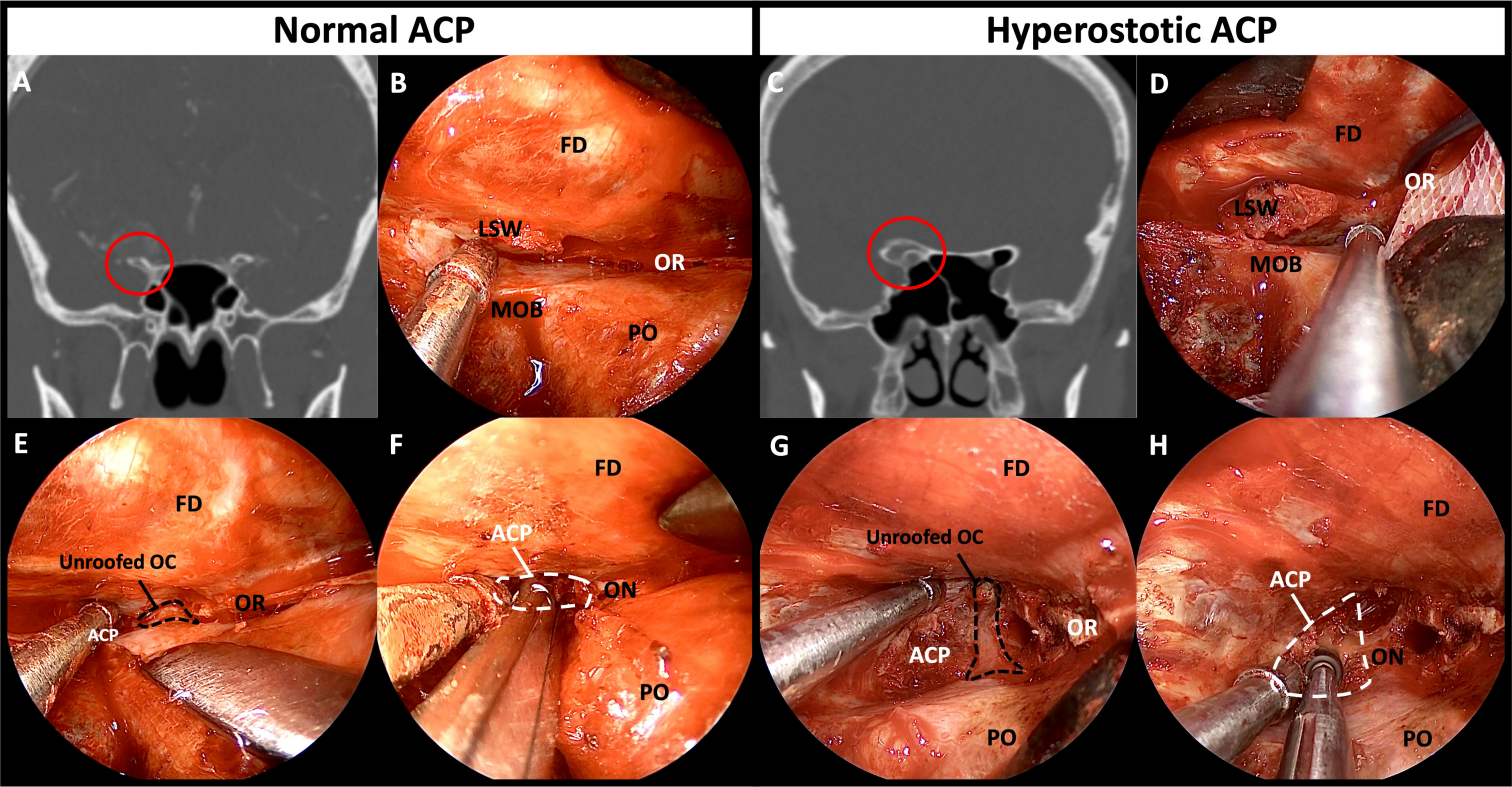
Comparison of bony structures between non-hyperostotic ACP and hyperostotic ACP. **(A)** Coronal image of preoperative CT of the patient with non-hyperostotic ACP. **(B)** Normal anatomy of the thin LSW and orbital roof. **(C)** Coronal image of preoperative CT of the patient with hyperostotic ACP. **(D)** Hyperostosis induced thickening of the LSW and the orbital roof. **(E)** Thin roof of the optic canal opened by fracturing or rongeuring using forceps. **(F)** The ACP removed by manipulation and fracturing the optic strut. **(G)** Drilling the bony roof of the optic canal using a small diamond drill tip for unroofing. **(H)** Drilling the ACP center and hyperostotic optic strut for facilitating removal of the ACP. ACP, anterior clinoidal process; CT, computed tomography; FD, frontal dura; LSW, lesser wing of sphenoid bone; MOB, meningo-orbital band; OC, optic canal; ON, optic nerve; OR, orbital roof; PO, periorbita.

The frontal dura was detached from the orbital roof until a good field of surgical view was obtained, and the location of the optic canal was confirmed. The roof of the optic canal (the anterior root of the ACP) should be carefully opened under the direct vision without causing damage to the optic nerve. Owing to the thinness of the bony roof of the optic canal of a nonhyperostotic ACP, the roof can be easily removed using a cutting forceps or can be fractured using a dissector or a curette. In the case of a hyperostotic ACP (common condition in ACP meningioma), the bony roof of optic canal should be carefully drilled using a small diamond tip to expose the optic canal dura (**Figure 5**). Potential thermal injury of the optic nerve by the heat generated in this process can be prevented by irrigation.

#### 3.2.4 Step 3

The ACP was circumferentially detached from the surrounding dura using a dissector. From the surgical view of the ETOA, the optic nerve is supero-medial, the 3^rd^ nerve was infero-lateral, and the clinoidal ICA was postero-medial to the ACP. The optic nerve and 3^rd^ nerve could be dissected from ACP under direct visualization. However, because the ICA was hidden by the ACP and the optic strut, extremely careful dissection of dura from the posterior surface of ACP was carried out to prevent an ICA injury.

#### 3.2.5 Step 4

The ACP gently twisted to fracture the optic strut (the posterior roof of the ACP) and the separation of all 3 supporting roots of the ACP from the adjacent bony structures was completed. For a hyperostotic ACP, drilling of the cancellous bone of ACP and optic strut make the ACP removal easier. Injury to the ICA, optic nerve, and 3^rd^ nerve from bone fragment should be avoided. Venous bleeding from the clinoidal space following to the ACP removal can be easily controlled using injectable hemostatic agents.

#### 3.2.6 Step 5

The ACP was pulled out without damaging the adjacent neurocritical structures. Simultaneously, the remaining adhesions along with the surrounding tissues were carefully dissected from the ACP. Microforceps should be used to remove any remnant bony fragments of the ACP.

### 3.3 Cadaver study

**Figure 6:**
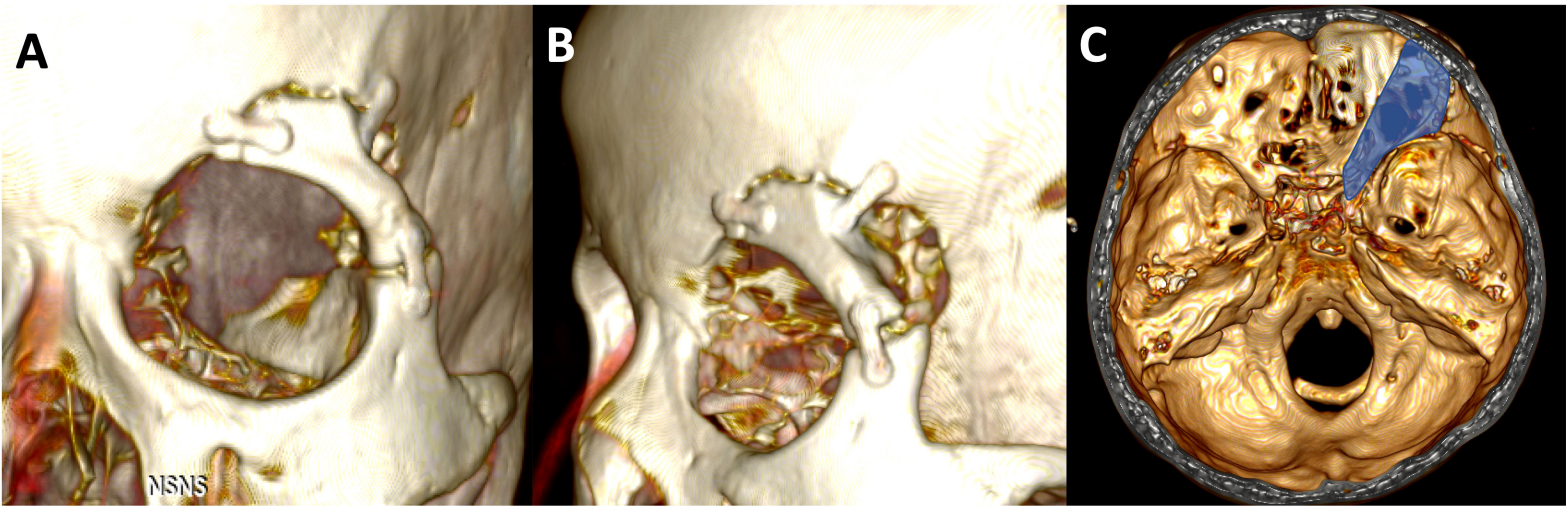
**(A, B)** 3D reconstruction images from postoperative CT scan of the patients underwent extradural anterior clinoidectomy using ETOA with SLOR osteotomy. Superior-lateral orbital rim was resected in one-piece and fixed firmly with miniplates. **(C)** The skull defect including ACP is showed as the blue highlighted area. ACP, anterior clinoidal process; ETOA, endoscopic transorbital approach; SLOR, superior-lateral orbital rim.

Extradural anterior clinoidectomy with a 5-step technique was successfully performed in all cadavers. Cutting the MOB and interdural dissection of lateral wall of CS was helpful to secure the lateral and posterior part of the ACP. However, anterior clinoidectomy could be achieved completely without this maneuver because the surgical direction in ETOA is parallel to the longitudinal axis of the ACP. The optic canal unroofing procedure could be safely performed without an injury to optic canal dura and optic nerve. As a result of Steps 1 and 2, 2 of the 3 roots of ACP were effectively disconnected and surgical space was secured for dissection between the ACP and surrounding structures. Existence of dura covering the critical neurovascular structure around the ACP can make it possible to detaching them from ACP without injury by careful dissection. After circumferential dissection of dura from ACP, the posterior root of ACP (optic strut) was fractured by gentle twist of ACP, followed by careful pulling out of ACP in one piece. In some specimens in which the resection of ACP could not be done in one piece, anterior clinoidectomy was performed in a piecemeal fashion using microforceps. The anterior clinoidectomy cound be achieved in all specimens without damage to adjacent structures. Adding SLOR osteotomy to conventional ETOA for the 5-step procedure of extradural anterior clinoidectomy could make the procedure safer and easier. With the ETOA with SLOR osteotomy, it would be easy to secure the surgical field and view because the vertical movement range could be increased and the retraction of the frontal dura was possible. This advantages of ETOA with SLOR osteotomy can facilitated the detachment of frontal dura from orbital roof, safe unroofing of optic canal, and easy manipulation of ACP (**Figure 6**).

### 3.4 Clinical implications

**Figure 7:**
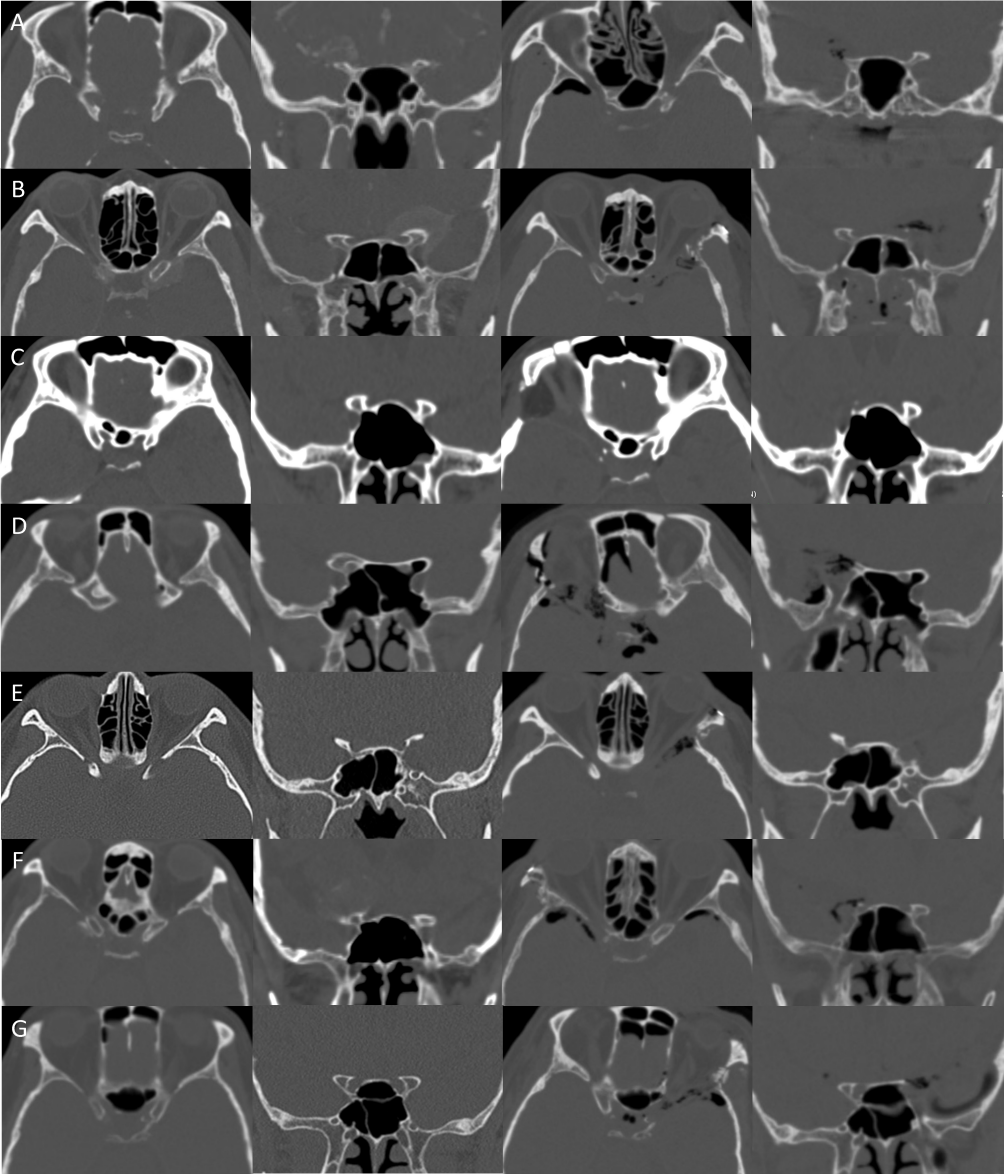
Clinical implication. **(A–G)** In seven cases, the stepwise extradural anterior clinoidectomy was completed. Complete removal of the ACP was confirmed with postoperative CT. CT, computed tomography.

**Figure 8:**
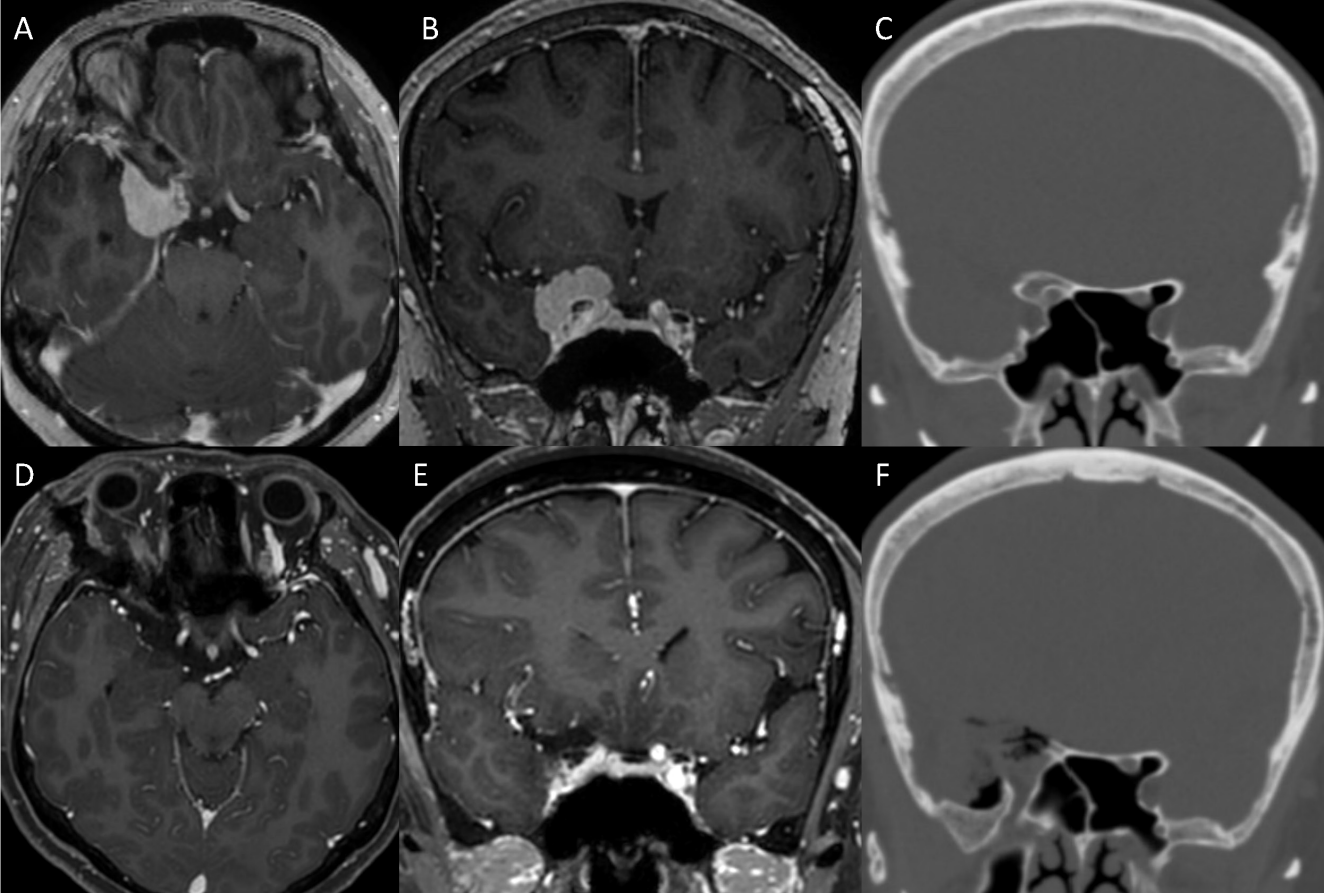
Illustrative case. **(A, B)** Preoperative MRI showing a mass in the central skull base, diagnosed as anterior clinoidal meningioma. **(C)** Preoperative CT showing the hyperostotic ACP. **(D–F)** Complete removal of the ACP and the tumor was confirmed with postoperative CT and MRI. ACP, anterior clinoidal process; CT, computed tomography; MRI, Magnetic resonance imaging.

The 5-step technique of endoscopic transorbital extradural anterior clinoidectomy was performed in 7 cases during the resection of brain tumor in the central skull base (**Table 1** and **Figure 7**). The anterior clinoidectomy was performed to enhance access to the lesion around the optic nerve and the ICA by improving the exposure of these structures. The average age of the patients was 56 years (range 36–72) years. The pathological result was ACP meningioma in 6 patients, and cavernous sinus meningioma in 1 patient (**Table 1**). In all cases, the anatomical landmarks were easily identified under the direct surgical view, and the stepwise extradural anterior clinoidectomy was completed. There was no complication involving cranial nerve injury, ICA injury, cerebral spinal fluid leakage (CSF), or infection in all cases. A case illustration is provided in **Figure 8** and **Video 2**.

**Table 1:** Clinical and demographic characteristics of patients who underwent stepwise endoscopic transorbital extradural anterior clinoidectomy.

Patient	Sex/Age (years)	Origin	Pathology	Skin incision	Approach	Extradural anterior clinoidectomy in a step-by-step fashion	EOR	Postop. complications
1	M/52	ACP	Meningioma	Below eyebrow	ETOA c SLOR	Complete	GTR	No
2	F/63	ACP	Meningioma	Below eyebrow	ETOA c SLOR	Complete	GTR	No
3	M/48	ACP	Meningioma	Eyelid	ETOA c SLOR	Complete	GTR	No
4	F/54	ACP	Meningioma	Below eyebrow	ETOA c SLOR	Complete	GTR	No
5	F/36	CS	Meningioma	Below eyebrow	ETOA c SLOR	Complete	STR	No
6	F/72	ACP	Meningioma	Below eyebrow	ETOA c SLOR	Complete	GTR	No
7	F/58	ACP	Meningioma	Below eyebrow	ETOA c SLOR	Complete	GTR	No

ACP, anterior clinoid process; CS, cavernous sinus; EOR, extent of resection; ETOA c SLOR, endoscopic transorbital approach with superior lateral orbital rim osteotomy; GTR, gross total resection; Postop., postoperative; STR, subtotal resection.

### 3.5 Illustrative case

A 54-year-old woman was admitted for headache and visual disturbance in the right eye. Preoperative magnetic resonance images (MRI) and CT scan demonstrated a 3cm anterior clinoidal meningioma in the right side with hyperostotic ACP. Taking the tumor size and its attachment with the adjacent neurovascular structures, the ETOA with SLOR osteotomy method was selected for complete removal of the tumor. Extradural anterior clinoidectomy was planned to expand surgical corridor and removal the hyperostotic bone for preventing recurrence, while achieving early control of the feeding vessels of the dura mater before tumor removal. A skin incision below the eyebrow was performed. After SLOR osteotomy, frontal-temporal craniotomy was performed. To achieve extradural anterior clinoidectomy, hyperostotic LSW (the lateral root of the ACP) was removed first. The MOB was cut and interdural dissection was performed to enhance the exposure of the ACP. After the dura in the medial frontal base was detached from the orbital roof and the ACP, the optic canal was identified. The hyperostotic bony roof of the optic canal was removed using an electric drill with a small diamond tip. After the unroofing of the optic canal was completed, the surrounding dura was detached from the ACP circumferentially using a dissector. Additional drilling in the center of the hyperostotic ACP was performed to facilitate manipulation of ACP. The ACP was held and twisted gently using forceps, and the optic strut fractured. The ACP was carefully pulled out after additional dissection from the surrounding adhesions. Finally, the ACP was safely removed in one piece. Complete anterior clinioidectomy was achieved through the stepwise manner. Since ethmoid sinus was opened during clinoidectomy, it was sealed with collagen matrix (Duragen) and absorbable fibrin sealant patch (TachoSil) to prevent CSF leakage. After the clinoidectomy, Simpson grade II resection of the tumor was carried out. Complete removal of the tumor and ACP were confirmed with postoperative MRI and CT. The patient recovered without any complications.

## 4 Discussion

The extradural anterior clinoidectomy technique proposed by Dolenc, has been widely accepted and used in classic transcranial skull base surgeries (16, 20, 32, 33). Anterior clinoidectomy can improve the exposure of structures in the central skull base including the optic nerve and the ICA, while promoting access to lesions around these structures (17, 24, 30, 34–36). The optic nerve can be visualized and decompressed earlier in the extradural anterior clinoidectomy by optic canal unroofing, which can reduce the probability of intraoperative injury to neurovascular structures during tumor manipulation (22, 23, 37, 38). In addition, extradural anterior clinoidectomy allows the devascularization of the tumor before resection by controlling dural feeding vessels (33, 39). Hyperostotic adjacent bones including the ACP which could be related to recurrence can also be removed by anterior clinoidectomy (20, 39). Hence, the extradural anterior clinoidectomy has been used in transcranial approach for treating lesions in the parasellar and pericavernous regions (40–42). Even though ETOA could also be used for the lesions in that region, anterior clinoidectomy through this approach was rarely performed until now due to technical and anatomical complexities. Removal of the ACP is difficult even with the transcranial approach because of its deep location in the central skull base and its proximity to vital neurovascular structures. In ETOA, moreover, the anatomical orientation around the ACP is confusing for neurosurgeons because of the unfamiliarity with the surgical view of the approach. Currently, there are only few studies of the surgical anatomy and technique for endoscopic transorbital anterior clinoidectomy, and no study about the clinical implication of this procedure in ETOA (2, 11, 43). To the best of our knowledge, this is the first technical study with clinical series suggesting this reproducible surgical technique and thus proving its clinical feasibility.

### 4.1 Advantages of the endoscopic transorbital extradural anterior clinoidectomy

The first advantage is that the transcranial extradural anterior clinoidectomy. The dura acts as a protecting barrier for the adjacent critical neurovascular structures and brain cortex, thus reducing the risk of injury to those structures (43, 44). Second, the direction of surgical corridor in the ETOA which is parallel to the longitudinal axis of the ACP is more feasible for the ACP removal (7, 31, 45). The direction to access the ACP in the classic transcranial approach is from lateral to medial, requiring incision of the MOB and peeling of the temporal dura from the lateral wall of the CS for sufficient exposure of its distal end (46, 47). This is mandatory for the safe resection of the ACP in transcranial approach (20, 48). However, the cadaveric study showed that complete extradural anterior clinoidectomy in ETOA was possible without this procedure because the direction of access can make the exposure of the distal end of the ACP possible while detaching the surrounding dura from it. Additionally, it requires the interdural dissection of only a small segment of the anterior part of the CS. Minimal invasion of the lateral wall of the CS can reduce venous bleeding and potential nerve damage caused by dura peeling from the CS lateral wall along the length of the ACP in the transcranial approach (48–50). This time-consuming procedure was minimized in our clinical cases without severe bleeding or nerve damage. Third, the three supporting roots of the ACP can be safely removed under the enhanced visualization using an endoscope which provides panoramic view with adequate magnification. The LSW (lateral root of the ACP) can be resected by routine frontotemporal craniotomy in the ETOA. After identifying the optic canal from below, the roof (anterior root of the ACP) can be directly visualized and opened using forceps or a drill. Drilling of the optic strut (posterior root of the ACP) is extremely risky during extradural anterior clinoidectomy because it lies beyond the ACP under the surgical view of transcranial approach. In the ETOA, it can be easily identified between the optic canal and superior orbital fissure because the optic strut lies medial to the ACP. It can then be dissected from the surrounding dura and drilled under the direct visualization using an endoscope of appropriate magnification. With these advantages, the EOTA can be applied to the central skull base lesions which need anterior clinoidectomy, in which the clinoidectomy can be performed in a relatively simple way. Considering the surgical trajectory of ETOA, the lesions which have an epicenter near ACP and occupy both middle and anterior cranial fossa, such as anterior clinoidal meningioma, can be amenable to this technique.

### 4.2 Avoiding complications

This technique has the disadvantage of extradural procedure including suboptimal visualization of key neurovascular structures around the ACP (21, 51, 52). The clinoidal ICA can get nearly blocked from the surgical view of the ETOA, because it lies posterior and medial to the ACP. Unlike the position of the optic nerve and 3^rd^ nerve which can be anticipated in the optic canal and SOF respectively, the ICA position can only be confirmed after anterior clinoidectomy. Accordingly, the dissection between the dura over the ICA and ACP can be risky during the endoscopic transorbital extradural anterior clinoidectomy. To prevent an ICA injury, careful dissection of the posteromedial margin of the ACP must be performed. Using the doppler can help to confirm the ICA location before dissection. The optic canal and SOF are easily identified in early stages of the procedure, hence, direct injury to the optic nerve and cranial nerves passing through the SOF is unlikely. To prevent cranial nerve injury, nevertheless, surgeons should avoid high power coagulation in proximity to these nerves despite them being covered with dura mater. In addition, thermal injury during the drilling of the ACP and the roof of optic canal should be avoided by frequent irrigation. Post the ACP removal, the clinoidal space is opened and it can be connected to the channels of the CS, which may cause massive venous bleeding. Although controlled using hemostatic materials, operators should be careful when using hemostatic material because overpacking could cause cranial nerve compression or an unfavorable chemical reaction.

Because the surgical field where the endoscopic transorbital extradural anterior clinoidectomy is carried out is deeper than the area for the routine bone work of ETOA, medial retraction of the orbital contents can be performed closer to the orbital apex. Since the orbit is a cone-shaped cavity, the optic and cranial nerves passing the SOF can be crowed in the narrow space of the orbital apex. Hence, deep retraction is likely to injure the cranial nerves by intraorbital pressure (53). Surgeons should place the retractor cautiously and pay close attention to the intraorbital pressure. The extended ETOA with lateral orbital rim osteotomy or SLOR osteotomy can also help to reduce the retraction of the orbital contents (11, 54).

The preoperative CT imaging should be meticulously inspected to understand the bony anatomy around the ACP for preventing the postoperative complications. Although usually constant, it can sometimes present anatomical variations, such as presence of a middle clinoid process, ACP pneumatization, interclinoid bridge, or variations of the optic strut shape, which in turn can increase the risk of surgical complications (26, 55). While the optic canal is being unroofed, ethmoid sinus can be opened at the medial part of the optic canal if excessively developed. To avoid an entry into ethmoid sinus, the medial limit of bone resection should be designated after meticulous inspection of the bony anatomy using preoperative CT scan. If the ethmoid sinus is opened during surgery, the opening should be elaborately sealed using fat, muscle patch, sealant, or artificial materials to prevent the CSF leakage and the infection.

### 4.3 Additional considerations for endoscopic transorbital extradural anterior clinoidectomy

The step involving identification and unroofing of the optic canal is key to safe and successful anterior clinoidectomy in the ETOA. This allows early decompression of the optic nerve during surgery, which can minimize the possible damage to the optic nerve during the subsequent steps (22, 38, 56, 57). The optic canal could be readily confirmed from below after detaching the periorbita from the orbital roof, while the grove whit the optic canal can be detected using a blunt hook dissector. For opening the optic canal, the frontal dura should be sufficiently detached and retracted from the orbital roof to secure the space for unroofing under the visualization of entire course of the optic canal. In a previous study, we realized that the structures constituting the ACP and the path of the optic nerve could be easily identified and accessed in ETOA with SLOR osteotomy which has superior surgical view of the frontal base, sphenoid ridge, and better vertical movement of the device compared to the conventional ETOA (11). Although extradural anterior clinoidectomy with conventional ETOA was accomplished in this cadaveric study, there was a limitation in manipulating the frontal dura because of the superior orbital rim. Additionally, since frontal lobe retraction may be required for extradural dissection around the ACP in the absence of sylvian splitting, the ETOA with SLOR osteotomy can be advantageous when a fixed retraction system is used (11). Therefore, when extradural anterior clinoidectomy in ETOA is required, ETOA with SLOR osteotomy offers simpler and safer procedure conditions. Hence, this technique was carried out for the clinical cases in this study to achieve a complete anterior clinoidectomy without complications.

A meningioma near the ACP can make it hyperostotic, thus making anterior clinoidectomy more complex (58, 59). In this case, optic canal unroofing, fracturing the optic strut, and pulling the ACP out can cause injury to the optic nerve and ICA, besides being time-consuming. The hyperostosis should therefore be checked before surgery using preoperative imaging study, and drilling should be carried out with care while the entire course of optic canal is being unroofed. To minimize the risk during manipulation of the optic strut and ACP, surgeons can additionally drill the hyperostotic ACP and optic strut until the thin cortical bone remains. Special caution should also be taken when removing the remaining cortical bones to avoid damage to the optic nerve and ICA.

In this study, only the extradural anterior clinoidectomy was applied, because intradural anterior clinoidectomy in the ETOA remains a limited method due to technical difficulties. In that method, delicate drilling skills are required to remove the ACP near critical neurovascular structures without the dural protection (60). Longer instruments are usually needed in the ETOA because of the deeper surgical field, which could disrupt fine control of drill tip and increase the probability of drill-induced injury to the exposed neurovascular structures (60–62). Furthermore, in contrast to transcranial approach, the frontal lobe of the brain is located superiorly in the surgical field of the ETOA which can get lowered by gravity, preventing access to the deeply located ACP. Difficult brain retraction and arachnoid dissection in the ETOA are additional barriers. Further anatomical and clinical studies are needed to propose the technique of intradural anterior clinoidectomy in the ETOA to validate its feasibility.

## 5 Conclusions

We designed a 5-step surgical technique for safe and efficient extradural anterior clinoidectomy through the ETOA and applied it to clinical cases to confirm its utility. This technique besides being safe, enhanced access to the lesion around the optic nerve and the ICA by improving the exposure of these structures. We believe endoscopic transorbital extradural anterior clinoidectomy could be the first step to expand the territory of the ETOA beyond the ACP to the frontal skull base.

## Data availability statement

The raw data supporting the conclusions of this article will be made available by the authors, without undue reservation.

## Ethics statement

The studies involving human participants were reviewed and approved by the cadaveric study committee of the Yonsei University of Medicine. The patients/participants provided their written informed consent to participate in this study.

## Author contributions

Conception and design: JM, JL, and KS. Acquisition of data: all authors. Analysis and interpretation of data: JM, JL, and KS. Drafting the article: JM, JL, KS. Critically revising the article: JM, JL, and KS. Reviewed submitted version of manuscript: all authors. Approved the final version of the manuscript on behalf of all authors: JM. Administrative/technical/material support: JM, JL, KS. Study supervision: JM.

## Funding

This work was supported by a faculty research grant from the Department of Neurosurgery, Yonsei University College of Medicine (JM) and by grants from the Ministry of Science, Technology and Information, Republic of Korea (Grant No.2021R1F1A105780111) (JL)

## Acknowledgments

We deeply appreciate Mr. Jun Ho Kim and Mr. Jong Ho Bang in the Surgical Anatomy Education Center of Yonsei University College of Medicine for their technical support. ‘SevEN’ is the ‘Severance Endoscopic Neurosurgery’ study group. This paper is the article from the SevEN.

## Conflict of interest

The authors declare that the research was conducted in the absence of any commercial or financial relationships that could be construed as a potential conflict of interest.

## Publisher’s note

All claims expressed in this article are solely those of the authors and do not necessarily represent those of their affiliated organizations, or those of the publisher, the editors and the reviewers. Any product that may be evaluated in this article, or claim that may be made by its manufacturer, is not guaranteed or endorsed by the publisher.
